# The PROMISE study: a clear promise for functional stress testing in patients with suspected coronary artery disease

**DOI:** 10.1007/s12471-015-0689-2

**Published:** 2015-04-21

**Authors:** E.E. van der Wall

**Affiliations:** Netherlands Society of Cardiology/Holland Heart House, Moreelsepark 1, 3511 EP Utrecht, The Netherlands

Over the years, considerable debate has arisen whether a pure anatomical test would suffice to demonstrate the significance of coronary artery disease (CAD) in patients suspected for CAD, and–more importantly–whether this anatomical information would have a bearing on clinical outcome [1, 2]. In particular, coronary computed tomography angiography (CTA) has been put forward as an optimal non-invasive anatomical imaging test to detect CAD in patients with stable CAD [3]. A number of studies has shown that CTA has a high sensitivity, reasonable specificity and an extremely high negative predictive value [4–10]. Several large-scale studies have shown that a strategy of CTA use in the emergency department is associated with faster discharge, as compared with standard care, without a significant difference in event rates [11–13]. However, the lack of evidence supporting CTA in randomised trials has also been mentioned. As a consequence, the relative impact of data from non-invasive anatomical testing versus functional testing on subsequent management and clinical outcomes is not fully known.

At the recently held conference of the American College of Cardiology (ACC), San Diego, California, 14–18 March 2015, the Prospective Multicenter Imaging Study for Evaluation of Chest Pain (PROMISE) trial was presented by Pamela S. Douglas, MD (Duke University School of Medicine, Durham, North Carolina, USA) and simultaneously published online in *The New England Journal of Medicine* [14]. The goal of the PROMISE trial was to evaluate anatomical testing using CTA compared with functional testing among low- to intermediate-risk patients with chest pain suspicious for CAD. The primary hypothesis of the study was that the clinical outcomes in patients assigned to anatomical testing with the use of CTA would be superior to those in patients assigned to functional testing.

A total of 10,003 low- to intermediate-risk patients with chest pain (mean age 61 years, 53 % female patients, 21 % diabetics) were randomised to evaluation with an anatomical strategy (*n* = 4996) versus a functional strategy (*n* = 5007). Patients randomised to an anatomical strategy underwent a 64-slice CTA, while patients randomised to a functional strategy underwent exercise electrocardiography (ECG), exercise imaging or pharmacological stress imaging. Among either group who underwent a functional test, 68 % underwent nuclear stress testing, 22 % underwent stress echocardiography and 10 % underwent exercise ECG. Duration of follow-up was a median of 25 months.

The primary outcome, all-cause mortality, myocardial infarction, hospitalisation for unstable angina, or major complication from a cardiovascular procedure occurred in 3.3 % of the anatomical testing group versus 3.0 % of the functional testing group (*p* = 0.75). Among low- to intermediate-risk patients with chest pain, anatomical testing with coronary CTA was not superior to functional testing. CTA was associated with an increased frequency of cardiac catheterisation; however, it was associated with a lower frequency of invasive catheterisation showing non-obstructive CAD. Anatomical testing was also associated with increased radiation exposure and a non-significant increase in total costs. In conclusion, in symptomatic patients with suspected CAD who required non-invasive testing, an initial strategy of CTA was not associated with better clinical outcomes than functional testing over a median follow-up of 2 years.

Simply stated, the PROMISE study suggests therefore that patients, suspected for CAD and undergoing CTA, do not have less risk of heart attack, dying or being hospitalised months later than those who take a simple treadmill test or other functional test. According to W. Douglas Weaver, MD, former president of the ACC, these findings should temper the enthusiastic use of CTA to screen patients with chest pain–it is not worth the added radiation and use of unnecessary heart catheterisations and stent implantations, which did nothing to improve the outcome of patients. Valentin Fuster, MD, current editor-in-chief of the Journal of the American College of Cardiology questioned if there could be a long-term benefit in direct visualisation using CTA. The study author, Pamela S. Douglas, said the research group plans to further investigate outcomes for different subgroups of patients to determine whether different groups might benefit from different testing approaches. In an accompanying Editorial [15], it was stated that the International Study of Comparative Health Effectiveness with Medical and Invasive Approaches (ISCHEMIA; ClinicalTrials.gov number, NCT01471522), in which randomised therapy (invasive versus medical, which is driven by the presence of extensive ischaemia on functional stress testing), will help answer this question.

So far, the PROMISE trial offers clear promise that functional stress testing provides at least similar information to CTA and–for economical and safety reasons–might prevail over CTA alone in patients with suspected CAD.
